# Data-driven concurrent nanostructure optimization based on conditional generative adversarial networks

**DOI:** 10.1515/nanoph-2022-0005

**Published:** 2022-05-09

**Authors:** Arthur Baucour, Myungjoon Kim, Jonghwa Shin

**Affiliations:** Department of Materials Science and Engineering, Korea Advanced Institute of Science and Technology, Daejeon 34141, South Korea

**Keywords:** color filter, fabrication tolerance, generative adversarial network, numerical optimization, structural color

## Abstract

Iterative numerical optimization is a ubiquitous tool to design optical nanostructures. However, there can be a significant performance gap between the numerically simulated results, with pristine shapes, and the experimentally measured values, with deformed profiles. We introduce conditional generative adversarial networks (CGAN) into the standard iterative optimization loop to learn process-structure relationships and produce realistic simulation designs based on the fabrication conditions. This ensures that the process-structure mapping is accurate for the specific available equipment and moves the optimization space from the structural parameters (e.g. width, height, and period) to process parameters (e.g. deposition rate and annealing time). We demonstrate this model agnostic optimization platform on the design of a red, green, and blue color filter based on metallic gratings. The generative network can learn complex M-to-N nonlinear process-structure relations, thereby generating simulation profiles similar to the training data over a wide range of fabrication conditions. The CGAN-based optimization resulted in fabrication parameters leading to a realistic design with a higher figure of merit than a standard optimization using pristine structures. This data-driven approach can expedite the design process both by limiting the design search space to a fabrication-accurate subspace and by returning the optimal process parameters automatically upon obtaining the optimal structure design.

## 1 Introduction

Numerical simulations are ubiquitous in the development of optical nanostructures, as they can quickly analyze design candidates before any actual fabrication and measurement, and are often used in conjunction with optimization methods such as particle swarm optimization [1, 2], genetic algorithm [3], [4], [5], deep Q-learning [6], or adjoint method [7], [8], [9], to refine the designs and significantly reduce the time of research and development. However, there can be some performance disparities between the simulated structures, with pristine shapes, and the real devices, with deformed profiles. These disparities can be significant for structures with small feature sizes such as visible-light metasurfaces.

Optical metasurfaces are composed of repetitive sub-wavelength structures arranged on a surface; they couple to the incident electromagnetic fields, thereby exhibiting effective properties that are not found in optical films made of natural materials [10]. The nature of the coupling depends not only on the constituent materials but also on the shape and size of the sub-wavelength motif. This morphology dependence opens a very large design space, and many previous proposals demonstrated an unprecedented performance and tunability in photonic applications such as color filters [11], [12], [13], [14], polarizers [15], [16], [17], [18], beam steering [19], [20], [21], absorbers [22], [23], [24], holograms [25], [26], [27], and many others [28].

However, this strong morphology dependence makes the design optimization much more time-consuming owing to the significantly increased dimension of the design space and leaves the metasurfaces vulnerable to the deformations from the fabrication process. This is particularly true for visible-light metasurfaces, of which the minimum feature size is often below 100 nm. Due to their small feature size, even sub-10-nm deformations may have a strong impact on performance. Therefore, if pristine shapes are assumed, then numerical simulations tend to overestimate the performance of the metamaterials and create a gap between the simulations and actual devices.

There are two approaches to solve this issue. Conventionally, when an optimal design is found, the fabrication process is optimized to obtain actual structures resembling the ideal shapes, leading to a second optimization process involving multiple fabrication and measurement cycles, which can be very time consuming, expensive, and without certitude to ever reach the ideal shape. Alternatively, the expected realistic shapes of the structure can be directly incorporated into the simulations, thereby increasing their fidelity. Studies have modeled deformations from fabrication processes such as etching and deposition processes [29], defects in micropillar cavities [30], and impact of particle defects in silicon photonic circuits [31], as well as statistically model dielectric metamaterials [32]. Such physical model-based simulations have been integrated in structure optimization loops [33, 34]. However, the simulation profiles are only as good as the model used to generate them. They may include some developer-bias or neglect some physical aspects of the fabrication process that are not captured by the model. Moreover, each fabrication step requires a specific model, thereby relying on domain expertise, and the use of different machines with the same fabrication process may lead to different results (e.g. a new top of the line evaporator against a heavily used and repaired one). Therefore, there is a need for a systematic modeling approach that can generate realistic profiles for the specific equipment and fabrication conditions used in the process.

In this study, we propose an alternative to these model-based realistic optimizations by introducing a data-driven concurrent optimization (DDCO) technique using conditional generative adversarial networks (CGAN) to learn accurate process-structure relations for the given fabrication processes. By learning from a training set, the generative neural network ensures that the simulation profiles are realistic for the given fabrication equipment and conditions. Therefore, this optimization platform explores the process and structural parameter space concurrently, thereby returning the optimal position in both spaces at the end of the optimization cycle.

**Figure 1:**
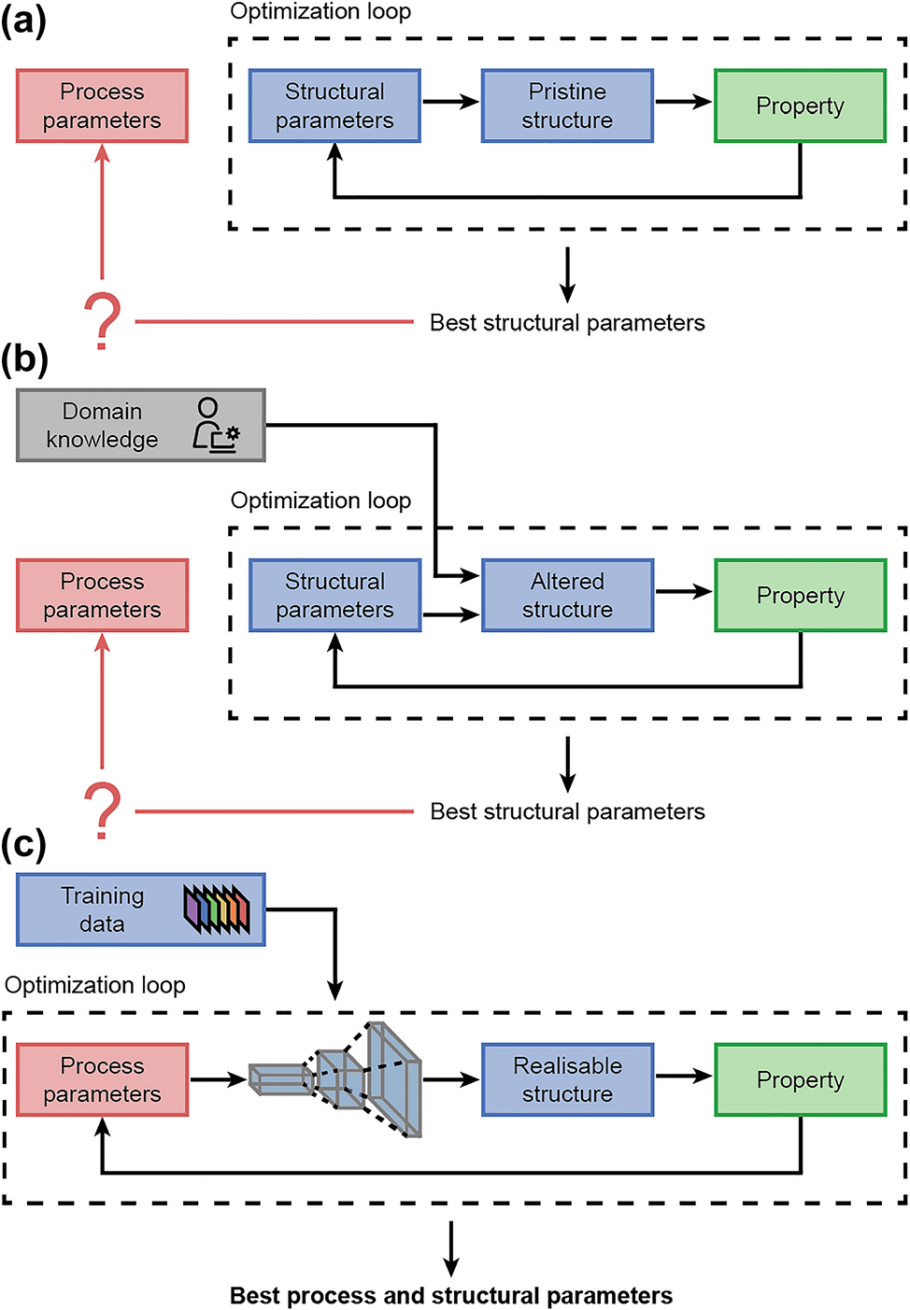
Main concept. (a) Standard optimization loop optimizes structural parameters assuming pristine structures (e.g. perfect rectangles). (b) Improved loop uses domain knowledge from researchers to realistically alter the simulation profile. (c) Proposed optimization loop optimizes the process parameters directly, and a neural network generates a realistic simulation profile used for optimization.

This is particularly useful for design optimization in three aspects. First, there is no need for preconceived knowledge about the process-structure relation. The generative model learns the mapping from the labeled training data. This data-driven approach limits the bias, simplifications, and arbitrary design decisions that may be taken when developing a physical model. It also makes this approach very general and easily applicable to different fabrication processes and training data, as there is no need to develop a new physical model. Second, it moves the design search space from the “structure” space (e.g., width and height) to the “process” space (e.g., electron-beam patterning width and evaporator deposition time), thereby returning the best fabrication process parameters directly upon the completion of the optimization, in addition to the structural shape, thereby eliminating the need for a time-consuming second fabrication optimization. Finally, this is a highly modular approach that can be fitted in a wide-range of optimization problems. One can replace the optimizer (e.g. genetic algorithm and particle swarm optimization), simulator (e.g. FDTD and RCWA), or generative method (e.g. CGAN or a different approach) while maintaining the same optimization workflow. A comparison between the usual optimization loops and the proposed approach is presented in Figure 1.

In this paper, we first present the use of CGAN to generate on-demand realistic simulation designs, and then demonstrate the complete optimization platform to design a red, green, and blue color filters based on silver gratings.

## 2 Conditional generative adversarial networks

A generative adversarial network (GAN) [35] is a neural network architecture used to train a generator network to produce an output similar to a training dataset. The generative model is pitted against a discriminative network that tries to identify if the data are real or generated by the model. These two networks are trained simultaneously, and ultimately the generator network can generate data that are undiscernible from the training set. GANs are commonly applied in image synthesis [36], which can be used to augment experimental data [37]. In photonics, GANs have been used to generate photonic crystals [38].

The main issue with the GAN architecture is that during the learning phase as well as in actual usage, the input of the generator network is random noise, and therefore the users have little control over the generated data. This is especially an issue in optimization problems, where output designs need to be generated in certain parts of the design space. To overcome this, an alternative architecture called conditional generative adversarial network (CGAN) [39] was developed, which conditions the generation process according to specific labels. In this architecture, the training set contains data to reproduce and their corresponding labels, and the generator network takes random noise and the desired labels as inputs. With such an architecture, the generator network can produce data similar to the subset of the training data with the given labels. The CGAN architecture is therefore useful for problems where realistic data need to be generated within specific conditions, and it has been used in applications such as super resolution [40], image-to-image translation [41], or text-to-image synthesis [42]. In photonics, CGANs have been used to predict the nanostructure shape for a desired reflection spectrum [43, 44]. Other examples include generating metagrating structures based on the desired wavelength and beam steering angle [45] and metasurfaces generating on-demand holograms [46]. While most of the previous publications that apply CGANs to photonic structures have focused on the inverse design to generate structures with on-demand optical properties, a recent study used CGAN to predict microstructure SEM images from laser-sintered alumina for various laser powers [47]. It generates structure images that are statistically similar to the training data, but the randomness of the images lends insignificance to the absolute location of the features.

In DDCO, the generative network generates realistic nanostructure binary profiles for a set of fabrication process parameters given as labels. This generative process should be able to replicate the defects occurring at specific locations with an almost pixel-to-pixel fidelity. Therefore, the use of CGAN in the presented approach is different from its use in inverse design, where it learns the mapping from property to structure to achieve on-demand properties. Here it learns the mapping from fabrication process to structure, enabling the optimization process to take place into fabrication parameter space and realistic structural parameter space concurrently. Moreover, the training goal is different from aforementioned generative methods in that usually generated data only needs to be statistically similar to the training data but there are some tolerances regarding the shape and position of the image features (e.g. generated SEM images needs to contain realistic features but their absolute position in the image is irrelevant). However, here, the generated profiles need to match as closely as possible the training data, without any leeway in term of position, orientation, etc. Ideally, the generated profiles should have a 1-to1 pixel correspondence with the training data.

As a conceptual demonstration, to predict the cross section of silver gratings made by three sequential fabrication processes, namely electron beam patterning, evaporator deposition, and thermal annealing, we consider a virtual fabrication process involving three continuous variable process parameters: patterning width, deposition time, and annealing time. These three parameters are combined with physical constraints, such as volume conservation over shape deformation and contact angle with the substrate, to generate plausible grating profiles. The suggested virtual experiment contains physical constraints, nonlinearity, and each structural parameter depends on all fabrication parameters, which makes it a reasonable example to demonstrate the potential of our approach to use CGAN to map process-structure relations. A complete description of the virtual experiment model is available in the Supplementary Information. This synthetic model can be replaced at any time by experimental data without changing the optimization workflow. The common goal is to train a CGAN so that it can generate simulation profiles similar to the training profiles for specific conditions to be used for the subsequent optimization process.

**Figure 2:**
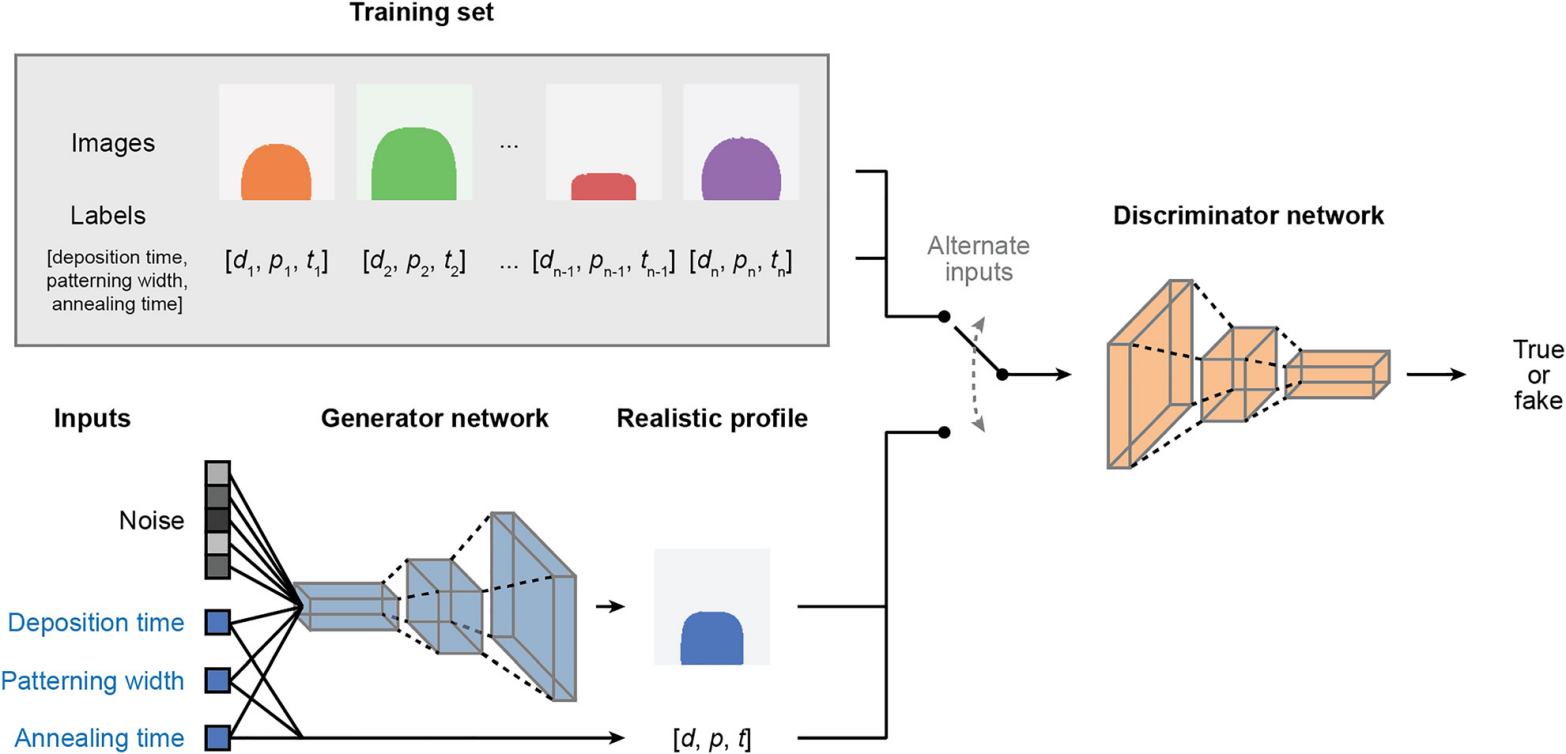
Training process. The training set is composed of both the images and their labels. The generator takes random noise and labels as its inputs and outputs a grating profile. The discriminator network alternates between receiving images and labels from the training set and generator network, and tries to determine if its input is real (from the training set) or fake (created by the generator network).

The training process is represented in Figure 2. The training set is composed of 10,000 computer-generated cross-sectional profiles corresponding to different patterning widths, deposition times, and annealing times. The training samples are randomly generated over the parameter space according to a uniform distribution. The training set contains both the binary images of the grating profiles and their process parameters as a label. The generator network takes the desired label and random noise as the input and delivers a 2D profile of the silver grating as the output. Then, either samples from the training set or produced by the generator network are passed to the discriminator network, which tries to determine if its input data is real, from the training set, or fake, generated by generator network. The two networks are trained concurrently and improve together: as the discriminator network gets better at identifying fake images, the generator network has to generate more realistic images. Details regarding the precise network architecture and training process can be found within the Supplementary Information.

**Figure 3:**
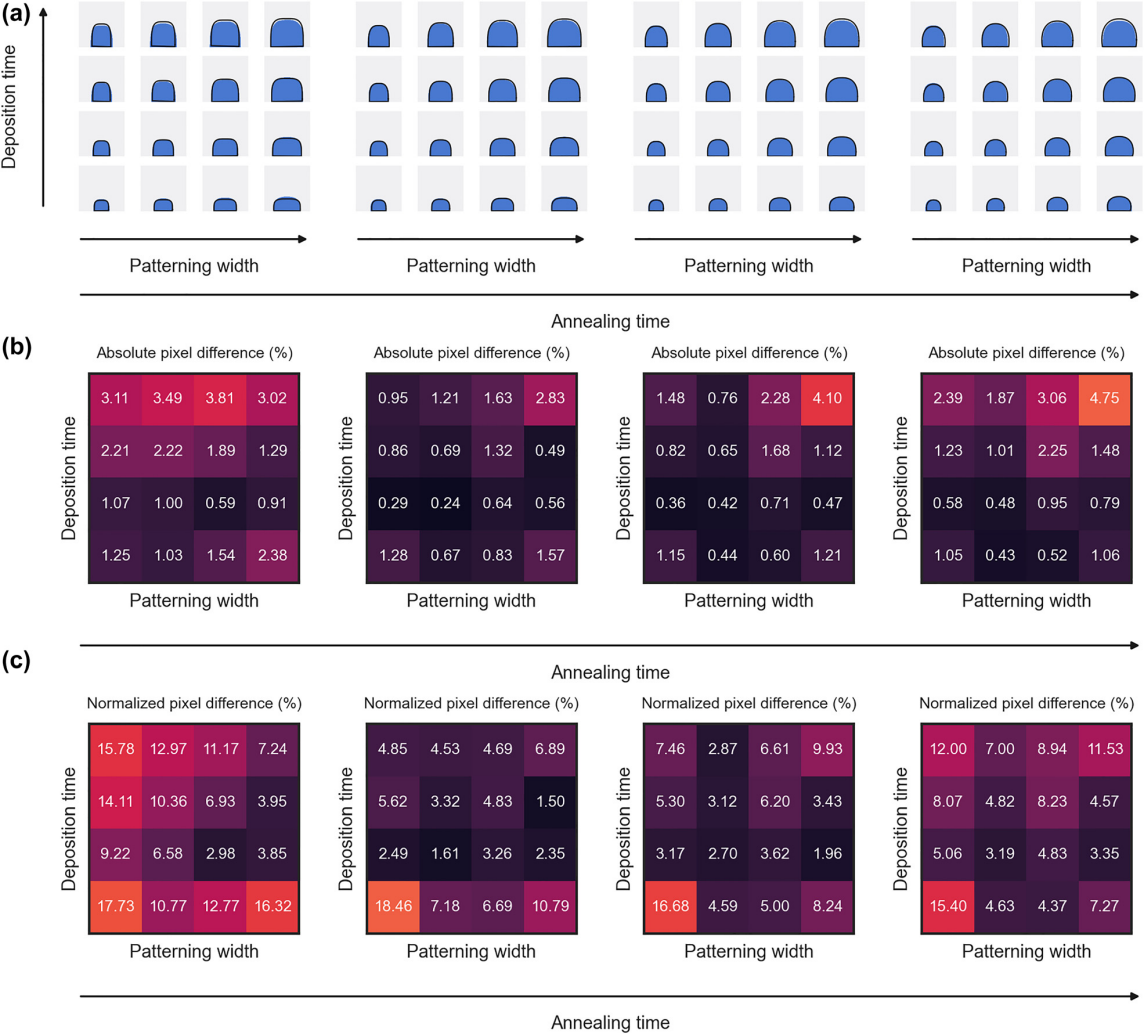
Generated profiles. (a) The generator network can produce structures, in blue, similar to the outputs of the virtual experiment, in black lines, over a large range of parameters. (b) Absolute pixel difference between the output of the neural network and the output of the virtual experiment process with same process parameters. (c) Pixel difference normalized by the area of the training profile (ground truth), highlighting the relative difference between the generated profile and the output of the virtual experiment.

The quality of the generated profiles is presented in Figure 3. After training, the generator network can produce on demand realistic profiles, similar to the outputs of the virtual experiment over the full continuous range of the parameter space. The difference between the generated and virtually fabricated profiles is measured by two metrics: the absolute pixel difference, counting the number of different pixels between the generated images and a fabricated profile with same parameters, and the normalized pixel difference, dividing the number of different pixels by the area of the training profile. While the absolute pixel difference gives information about the training quality and how close the produced images are, the normalized pixel difference gives information about the relative difference in grating cross-section area and, therefore, the potential difference in optical behavior. From the measured values, the CGAN training was successful, exhibiting an absolute pixel difference lower than 5% for all tested conditions, but the profiles generated near the boundaries of the parameter space (e.g. small patterning width and deposition time) shows a large normalized pixel difference, and therefore risk to show different performance than the fabricated structures. For this reason, the generator network will only be used in a slightly restricted parameter space, to ensure similar optical response between the generated and training profiles.

### 3 Application to color filter design

**Figure 4:**
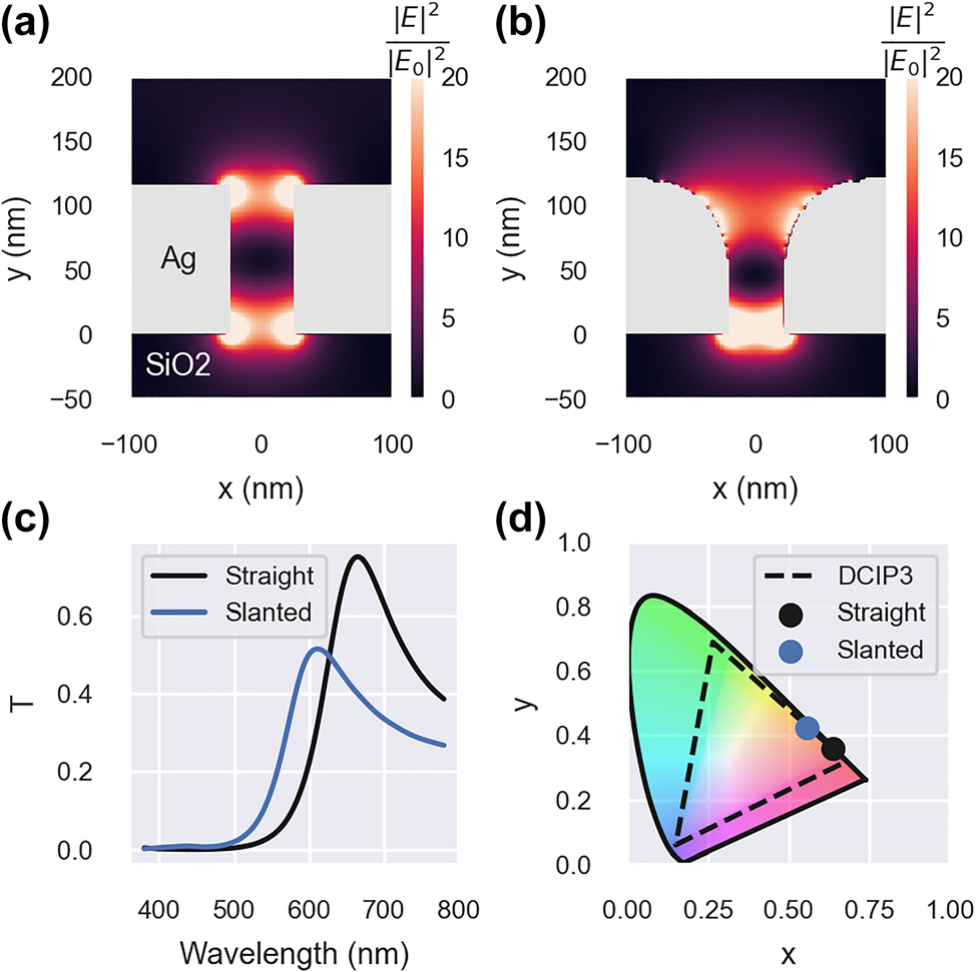
Impact of wall quality. (a) Magnitude of the electric field at the resonance wavelength (664 nm) in a grating structure with straight walls. (b) Magnitude of the electric field at the resonance wavelength (605 nm) in a grating structure with slanted walls. (c) Transmission spectra of straight and slanted walls simulations. (d) Perceived colors assuming a D65 illuminant and projected in the CIE 1931 color space chromaticity diagram.

Subwavelength metallic gratings can exhibit transmission resonances via two mechanisms: the coupled surface plasmon polaritons or waveguide resonances within the slits [48]. The latter has the advantage of showing a relatively low angular dependency and high transmittance [49]. However, since the resonance happens in the slit, it is strongly dependent on the wall quality of the gratings. This is illustrated in Figure 4, where we simulated the behavior of silver gratings (200 nm period, 115 nm height, and 150 nm width) embedded in silicon dioxide using finite-difference time domain simulations. The wall deformation altered the electric field distribution at the resonance, thereby affecting performance. The slanted wall simulation showed a lower maximum transmission coefficient and a resonance shift, leading to a noticeable change in the transmitted color. Therefore, to avoid overestimating the performance of such gratings, it is important to run the simulations with realistic profiles. Herein, the subwavelength metallic gratings are a good example to demonstrate the proposed optimization platform.

**Figure 5:**
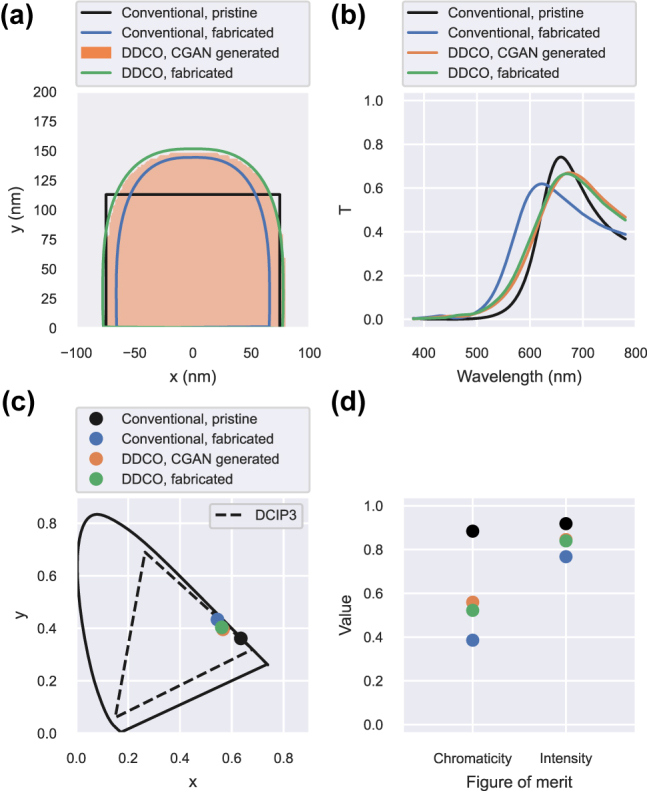
Optimization results. (a) Cross section of the different structures used in the optimization loops (pristine for conventional optimization and CGAN generated for DDCO) and the corresponding profiles fabricated by virtual experiments. (b) Transmission spectra. (c) Transmitted colors assuming a D65 illuminant source compared to the DCIP3 color space (dashed black line). (d) Figure of merit (FOM) of the different structures depending on the optimization.

As a demonstration, we designed a transmission-mode red color filter, and defined a figure of merit (FOM) that considered the maximum of transmission at the relevant wavelengths (intensity) and the purity of the perceived color (chromaticity). The specific FOM definition is available in the Supplementary Information. We conducted two optimizations: a conventional optimization using pristine shapes, with vertical walls and sharp corners, and another using the DDCO platform using a CGAN to generate realizable grating structures based on the fabrication conditions. During the CGAN-based optimization, the annealing time variable was set to a fixed value that represented a consistent fabrication process. The two optimizations used the same simulator, that is, the finite-difference time-domain (FDTD) method by Lumerical [50], and optimizer, that is, particle swarm optimization, but differed in how the simulation profiles were generated, thereby exploring different designs spaces, which led to different optimization results. The cross sections and performances of the optimized structures are presented in Figure 5.

The conventional optimization led to a pristine structure with a high transmission at the relevant wavelengths and good chromaticity, resulting in high FOMs. However, for these parameters, a significantly poor performance level of the realistic gratings was achieved through the virtual experiment. This illustrates that the conventional optimization technique assuming pristine structures may overestimate the performance of the actual devices with imperfect shapes. In comparison, DDCO led to an optimal realistic structure with a different set of structural parameters, most notably being the thicker gratings. The FOM of the fabricated design increased by 60.3 and 14.1% with respect to its chromaticity and intensity, respectively. It supports the idea that DDCO can find a more adequate optimum for the given training data, compared with conducting an optimization with ideal structures and expecting the performance level to be maintained when moving to realistic designs or experiments.

Both optimizations led to gratings as wide as permitted by the parameter space, thereby minimizing the width of the slits. However, the two optimized designs differ in height. The CGAN-based optimization led to thicker gratings. This change in the optimal parameters may be related to the curvature found in the training data. Deforming the edges of the gratings reduces their effective length, and therefore the realistic structures need to be thicker than the pristine gratings with perfectly straight walls to achieve the same resonance wavelength. This behavior may be observed in Figure 4, where the waveguide resonance is blue-shifted when the edges of the gratings were rounded.

By learning the expected shape for the given fabrication parameters, the generator network created grating profiles similar to those from the training data and reproduced the expected blue-shift of the resonance. Therefore, the CGAN-based optimization occurred in a more accurate design space than the conventional optimization and was able to find a structure with better performance than conducting ideal simulations and using the ideal fabrication parameters to generate realistic designs.

**Figure 6:**
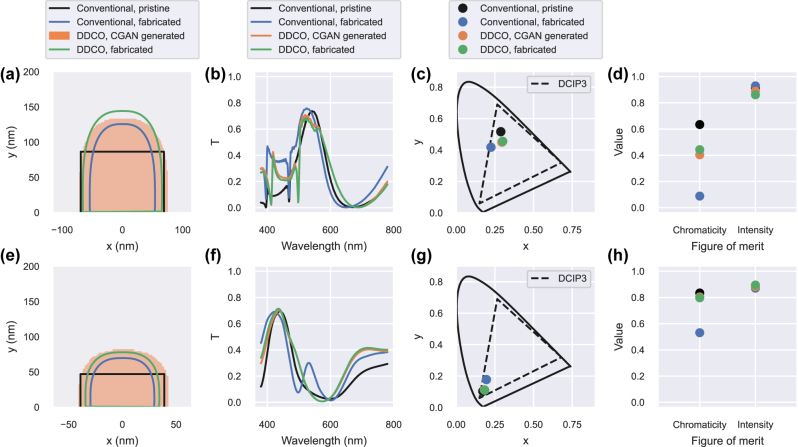
Waveguide-grating optimization results. (a, e) Cross section of the different structures used in the optimization loops (pristine for conventional optimization and CGAN generated for DCCO) and the corresponding profiles fabricated by virtual experiments for green and blue optimizations. (b, f) Transmission spectra. (c, g) Transmitted colors assuming a D65 illuminant source compared to the DCIP3 color space (dashed black line). (d, h) Figure of merit (FOM) of the different structures depending on the optimization.

For completeness, additional optimizations were conducted to design green and blue color filters, with the data available in the Supplementary Information. The DDCO optimization maintained small discrepancy between the CGAN generated profiles and the virtually fabricated structure. However, these optimizations did not produce good color filter designs, due to limitations of the structure and parameter space. Therefore, we introduced a more complex design based on a double-layer resonant waveguide-grating structure [51] consisting of a ZnS high-dielectric layer (*n* = 2.4) under the silver metallic grating to achieve proper green and blue results. This supplementary degree of freedom and new resonance mechanism enabled the optimization process to find structures with good transmission and color purity. The optimization results are presented in Figure 6, and the red color filter optimization with that structure are available in Supplementary Information. Due to the dimensions of the gratings, thin and high and for green filter and thin and small for the blue filter, we observe some difference in grating profiles between the data generated by the CGAN and the corresponding virtually fabricated profile. This is related to the training quality of structures on the fringe of the parameter space and particularly small structures, as discussed from Figure 3, where a few different pixels do not affect much the overall image (absolute pixel difference) but strongly affect the relative size difference between generated profile and the corresponding fabricated profile (normalized pixel difference). However, there is still a good agreement between the CGAN generated and virtually fabricated data. Moreover, DDCO in both cases lead to fabricated profiles with higher figure of merits than running conventional particle swarm optimization with pristine structure and using these parameters to achieve a realistic structure.

## 4 Conclusions

In summary, we introduced a data-driven approach to optimization that leverages training data to learn the process-structure relation using generative neural networks. We showed that a CGAN could be trained to generate realistic grating profiles similar to those from the training data based on a virtual fabrication process mimicking the electron beam patterning width, evaporator deposition time, and annealing time. The generated gratings reproduced the dimensions and deformations found in the training data accurately over a continuous parameter space. We applied this technique to optimize a red color filter based on silver gratings and green and blue filters based on a waveguide-grating structure. We found that the presented DDCO platform could find better fabrication parameters than if we conducted a conventional optimization using pristine structures and used these parameters to get a realistic sample through the virtual experiment process. By learning the expected deformations from the training samples, the CGAN gave a more realistic characterization of the optimization space and helped the optimization algorithm to find a more accurate optimum.

This data-driven approach to optimization does not require any preconceived knowledge or assumption about the process-structure relation and moves the design space from the structural parameter space to the process parameter space, thereby removing the need to optimize the fabrication process once the best structure is found, and directly returns the optimized fabrication and structural parameters. This is a general approach that can be applied to a wide range of fabrication processes and optimization loops. DDCO is an alternative to the model-based realistic optimizations, which uses a physical model to enhance the realism of the simulations. However, we believe that both approaches have their own merits. On one hand, the model-based approach is more transparent and can explain the physical mechanisms at play, while requiring domain expertise to build the model and potentially being biased or incomplete. On the other hand, the presented data-driven approach can be applied to any fabrication process without domain expertise and provides a model tailored to the specific equipment used, while relying on the quality of the training data.

Recent works have demonstrated the use of CGANs in the inverse design of photonic structures, generating structures with desired optical properties. Here, we presented how they can fit into a common forward optimization workflow, generating realistic structures based on the fabrication parameters. These two approaches are complementary sides of the process-structure-property relationships, and we can expect more applications as experimental datasets become available.

## Supplementary Material

Supplementary Material
