# Supplementary material for: Data-driven concurrent nanostructure optimization based on conditional generative adversarial networks
Source: Nanophotonics. 2022 May 9;11(12):2865–73. doi: 10.1515/nanoph-2022-0005 (PMC11501161; doi:10.1515/nanoph-2022-0005)
Supplement: Supplementary file 1 — Supplementary Material [file j_nanoph-2022-0005_suppl.docx]

Arthur Baucour, Myungjoon Kim, and Jonghwa Shin

**Supplementary material for: “Data-driven concurrent nanostructure optimization based on conditional generative adversarial networks”**

1. Data generation
   1. Overview

We use a semi-physical model of the morphological change of silver nanowire, described in Figure S1. We assume that the wire remains as a uniform wire and does not agglomerate into an array of particles. The main objective of this model is to provide a simple but semi-realistic model of morphological change of the wire cross-section upon annealing, to be used in generation of training database for CGAN. The model is not based on accurate microscopic physical processes but implement non-linear relations and physical constraints such as volume conservation and contact angle with the substrate. Hence, the model implement realistic behaviors and is not trivial to learn for the neural network.


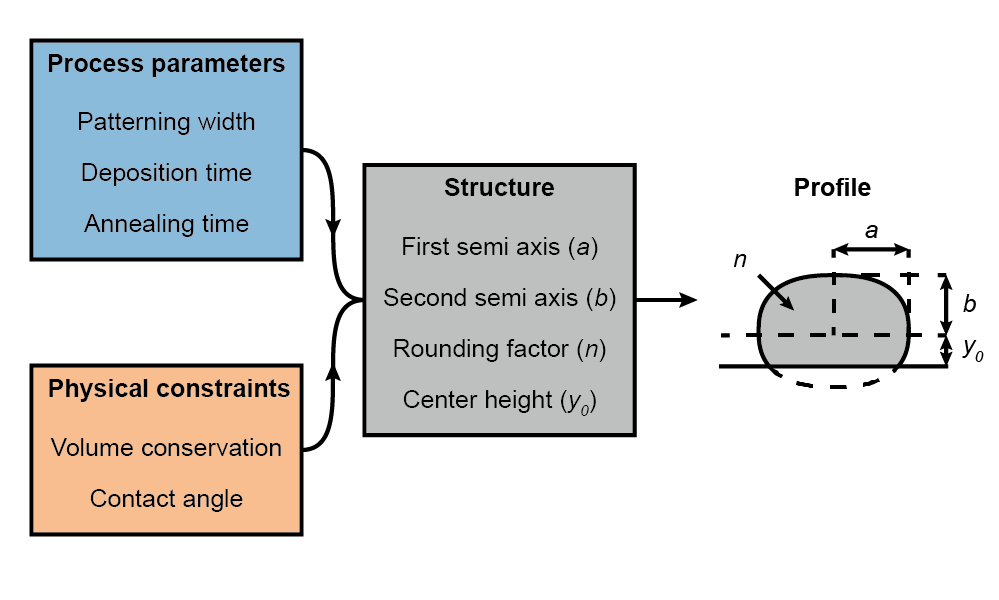


**Figure S1:** Data generation overview. The training samples were gathered by conducting virtual experiments taking into consideration the patterning width, deposition time, annealing time, and physical constraints such as volume conservation and contact angle.

- 1. Initial shape

The initial shape is assumed to be an hyperellipse defined by

$$\left| \frac{x}{a\left( 0 \right)} \right|^{n\left( 0 \right)}+\left| \frac{y-y_{0}\left( 0 \right)}{b\left( 0 \right)} \right|^{n\left( 0 \right)}=1,$$

With

$$\left\{ \begin{aligned} a(0)=initial width/2 \\ b(0)=initial thickness \\ y_{0}\left( 0 \right)=0 \end{aligned} \right.$$

Such structure can achieve a perfect rectangular shape, if $n\left( 0 \right)=\infty$, or make a structure with rounded angles, if $n(0)$ is a finite value.

- 1. Final Shape

The final shape is assumed to be a truncated circle:

$$\left| \frac{x}{a\left( \infty\right)} \right|^{n\left( \infty\right)}+\left| \frac{y-y_{0}\left( \infty\right)}{b\left( \infty\right)} \right|^{n\left( \infty\right)}=1,$$

With $n\left( \infty\right)=2$, $a\left( \infty\right)=b(\infty)$. We also assume that the contact angle of the final structure is a constant $\theta_{c}>\pi/2$. For example, gold on 3 nm chromium adhesive layer on quartz has $\theta_{c}\approx128^{\circ}$. We determine the values of $a(\infty)$ and $y_{0}(\infty)$ based on the constant volume and contact angle.

For the perfect rectangle case, the initial cross sectional area, $\mathcal{A}(0)$, is simply $2a\left( 0 \right)b(0)$. For a more general case of a rounded initial shape with a finite $n(0)$, the cross sectional area of the hyperellipse is determined by

$$\mathcal{A}\left( 0 \right)=2a\left( 0 \right)b\left( 0 \right)\frac{\left[ \Gamma\left( 1+\frac{1}{n\left( 0 \right)} \right) \right]^{2}}{\Gamma\left( 1+\frac{2}{n\left( 0 \right)} \right)},$$

In which Γ is the gamma function.

The final cross section area is that of a truncated circle. The untruncated region has a central angle of $2\theta_{c}$ and the truncated region an angle of $2\left( \pi-\theta_{c} \right)$. Therefore, the total area is given by

$$\mathcal{A}\left( \infty\right)=\theta_{c}a\left( \infty\right)^{2}+\cos\left( \pi-\theta_{c} \right)\sin\left( \pi-\theta_{c} \right)a\left( \infty\right)^{2}$$

$$\mathcal{A}\left( \infty\right)=\left( \theta_{c}-\frac{1}{2}\sin\left( 2\theta_{c} \right) \right)a\left( \infty\right)^{2}$$

As the area should be conserved, we have

$$a\left( \infty\right)=\sqrt{\frac{2a\left( 0 \right)b\left( 0 \right)}{\theta_{c}-\frac{1}{2}\sin\left( 2\theta_{c} \right)} \frac{\left[ \Gamma\left( 1+\frac{1}{n(0)} \right) \right]^{2}}{\Gamma\left( 1+\frac{2}{n\left( 0 \right)} \right)}} ,$$

We can also determine the final center height, given by

$$y_{0}\left( \infty\right)=-a\left( \infty\right)\cos\left( \theta_{c} \right)$$

Hence, we determined the final shape, a circle of radius $a\left( \infty\right)$ and center height $y_{0}\left( \infty\right)$.

- 1. Transitional shapes

For the temporal transition from the initial shape to the final shape, we can consider many different choices if we do not rigorously consider the actual microscopic physical processes involved. As a simple model, we adopt a hyperbolic cotangent function for $n(t)$ and an exponential saturation function for the aspect ratio, $a\left( t \right)/b\left( t \right)$, and the elevation, $y_{0}(t)$. In other words,

$$\left\{ \begin{aligned} n\left( t \right)=2\coth\left( \frac{t+t_{0}}{\tau_{1}} \right) \\ \frac{a\left( t \right)}{b\left( t \right)}=\frac{a\left( 0 \right)}{b\left( 0 \right)}\exp\left( -\frac{t}{\tau_{2}} \right) \\ y_{0}\left( t \right)=y_{0}\left( \infty\right)\left[ 1-\exp\left( -\frac{t}{\tau_{2}} \right) \right] \end{aligned} \right.$$

In which $t_{0}$ accounts for the initial rounding (i.e., $n\left( 0 \right)=2\coth(t_{0}/\tau_{1}$) and $\tau_{1}$ and $\tau_{2}$ are time constants that we need to specify. Once $n\left( t \right)$, $a\left( t \right)/b\left( t \right)$, and $y_{0}\left( t \right)$ are found, the exact shape at time t can be found by considering the volume conservation. The cross sectional area at time $t$ can be calculated numerically for different choices of $a(t)$ and the correct choice of $a(t)$ is selected such that the area remains conserved.


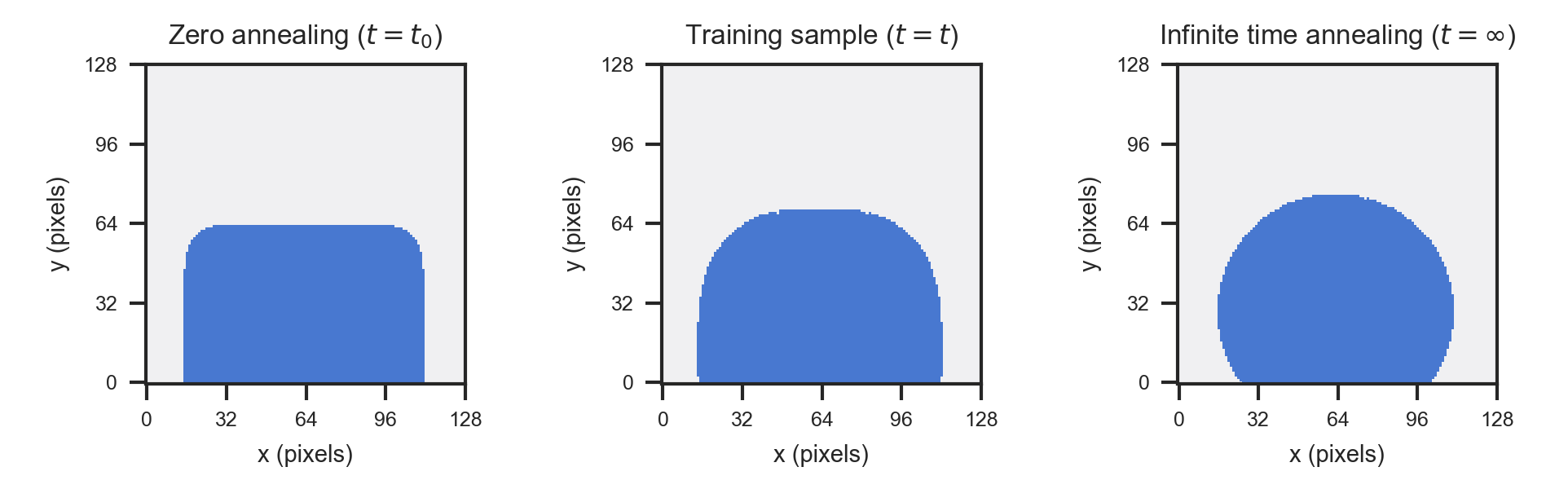


**Figure S2:** Virtual experiment modeling.

1. Neural network
   1. Architecture

The neural network architecture is presented in Figure S3. It is based on the DCGAN architecture^[[1]](#footnote-1)^, adapted to the different image resolution and conditional inputs.


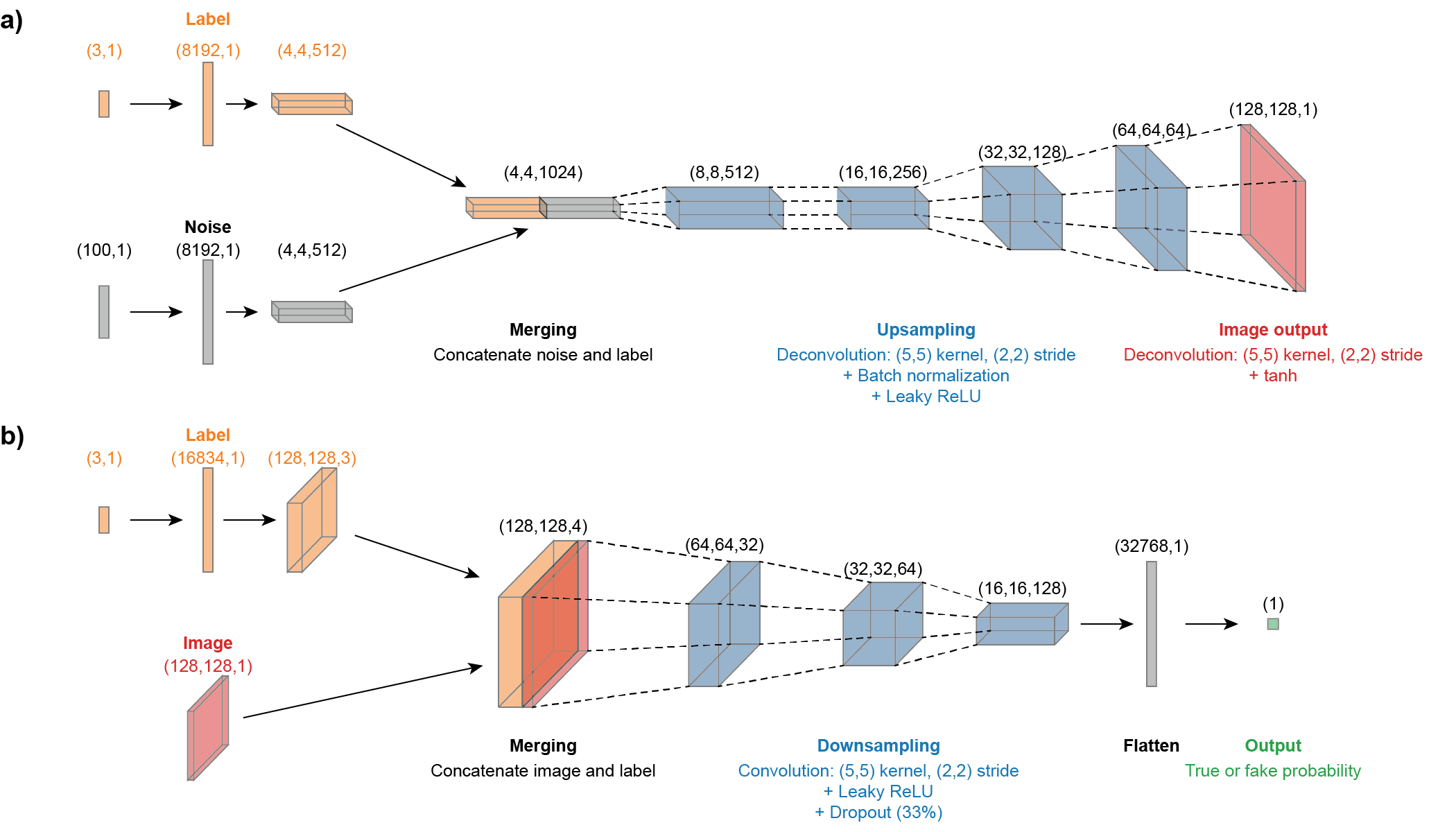


**Figure S3:** Network architecture. (a) Generator. (b) Discriminator.

- 1. Training parameters

The network was implemented in TensorFlow 2.1.0 and trained on a desktop computer with a NVIDIA GTX 2080 Ti graphics card, relying on CUDA to accelerate the training process. Training parameters are summarized in Table S1.

**Table S1:** Training parameters

| Training parameter | Value |
| --- | --- |
| Batch size | 8 |
| EPOCHS | 2000 |
| Training samples | 10000 |
| Loss function | Binary cross entropy |
| Optimizer | Adam, learning rate=1e-4 |

1. Particle swarm optimization
   1. Figure of merit

For color filter application, we aim to optimize both the intensity and chromaticity. Explicit definition of individual components of the figure of merit (FOM) are given in Table S2 and represented in Figure S4. Both components are then combined in an overall FOM according to the following equation:

$$FOM=\left( FOM Chroma \right)^{3}\times\left( FOM Intens \right)^{1}$$

Such balance emphasize the importance of the chromaticity and favor designs with better color purity.


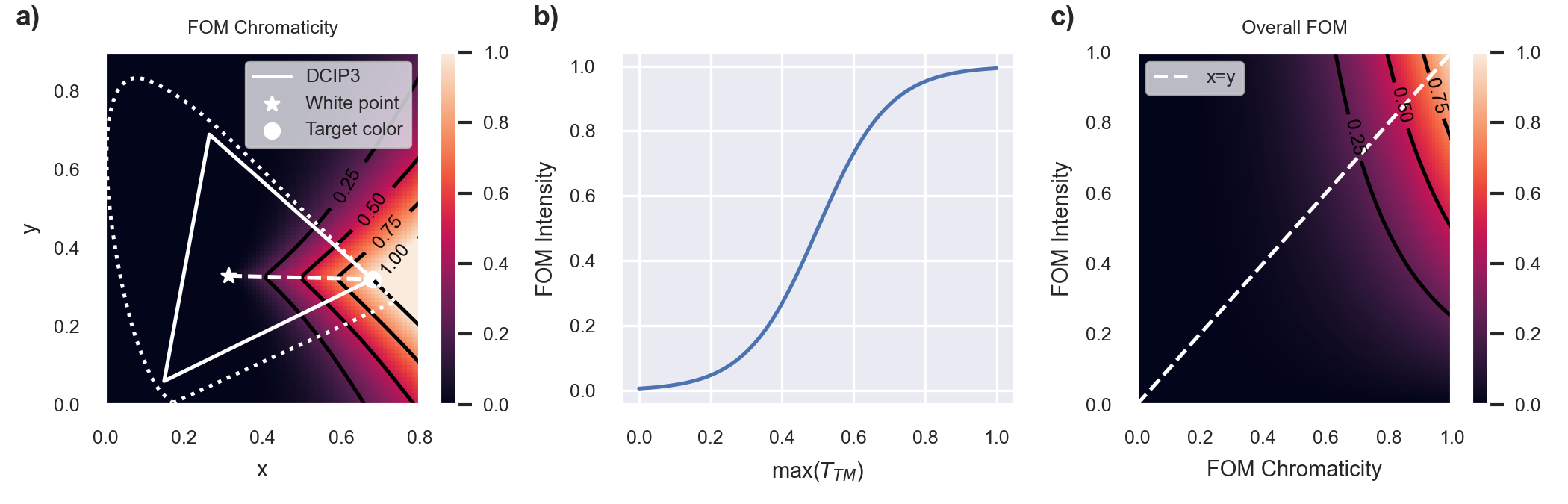


**Figure S4:** Components of the figure of merit. a) Chromaticity; b) Intensity c) Overall FOM depending on the value of its individual components.

**Table S2:** Individual components of the figure of merit

| Constraint | Equation | Parameter | Description |
| --- | --- | --- | --- |
| Intensity | $f\left( x \right)=\frac{L}{1+\exp\left( -k\left( x-x_{0} \right) \right)}$ | $x=\max\left( T_{TM} \right)$ | TM transmission maximum over the visible range |
|  |  | $L=1$ | Maximum value |
|  |  | $k=10$ | Steepness of the curve |
|  |  | $x_{0}=0.5$ | Midpoint |
| Chromaticity | $f\left( \vec{x} \right)=\frac{\left\vert\vec{x} \right\vert}{\left\vert\vec{t} \right\vert}\exp\left( -\theta^{2} \right)\left( 1-\sin\vert\theta\vert\right)$ | $\vec{x}=(x,y)$ | Coordinates in xyY chromaticity diagram |
|  |  | $\vec{t}=(x_{Target},y_{Target})$ | Target chromaticity |
|  |  | $\theta$ | Angle between $\vec{x}$ and $\vec{t}$ |

- 1. Parameter space

**Table S2:** Parameter space for the optimization of grating designs. The period is fixed at 200 nm.

| Parameter | Minimum (nm) | Maximum (nm) |
| --- | --- | --- |
| Grating’s width | 50 | 150 |
| Grating’s thickness | 50 | 200 |

**Table S3:** Parameter space for the optimization of waveguide-grating designs. The grating’s width is 0.65 times the period.

| Parameter | Minimum (nm) | Maximum (nm) |
| --- | --- | --- |
| Period | 75 | 230 |
| Waveguide’s thickness | 50 | 600 |
| Grating’s thickness | 50 | 200 |

- 1. Optimization process

The optimization process follows a standard particle swarm optimization algorithm, using 30 particles exploring the design space for 60 generations to find the structure with the highest figure of merit. The convergence of the swarm toward the best structure is presented in Figure S5.


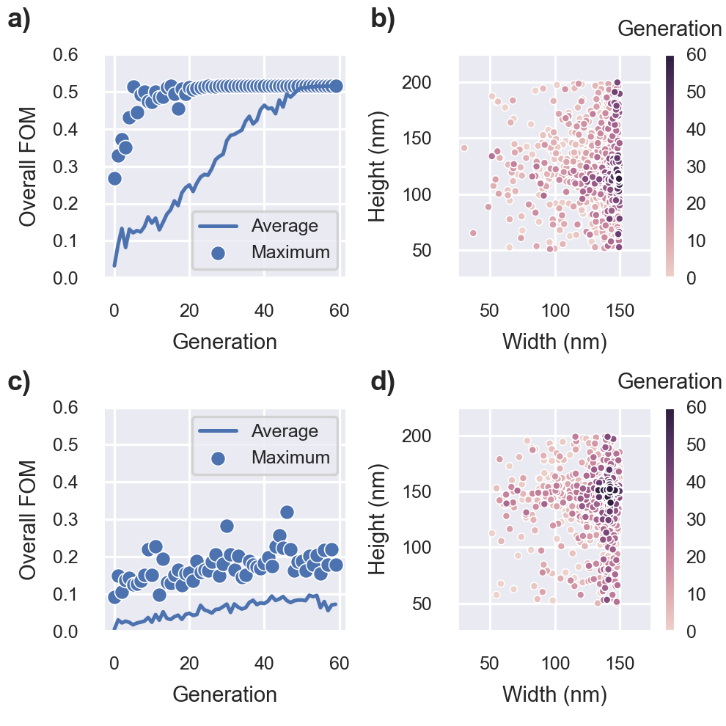


**Figure S5:** Swarm behavior. (a) Evolution of overall FOM for a conventional optimization with pristine structures; (b) Particle movements in the design space over the generations for a conventional optimization; (c) Evolution of the overall FOM for a CGAN-based optimization using simulations profiles generated by the neural network; (d) Particle movements in the design space over the generations for a CGAN-based optimization.

1. Waveguide resonance
   1. Angular dependence


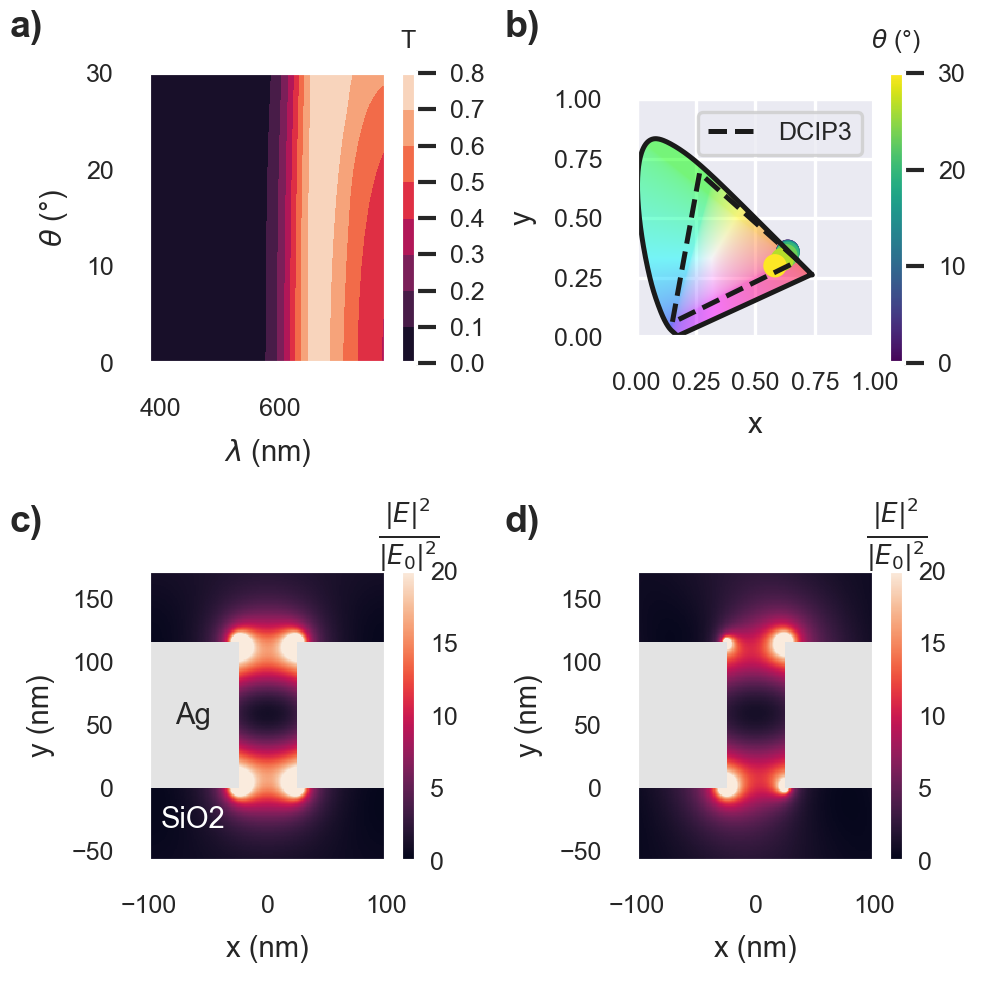


**Figure S5:** Information about angular dependency. a) TM transmission as a function of the incident angle. b) Perceived chromaticity in the CIE1931 color space chromaticity diagram, assuming a D65 illuminant. c) Electric field distribution at the resonance wavelength at normal incidence illumination and d) at 30 degree incidence.

1. Extended results


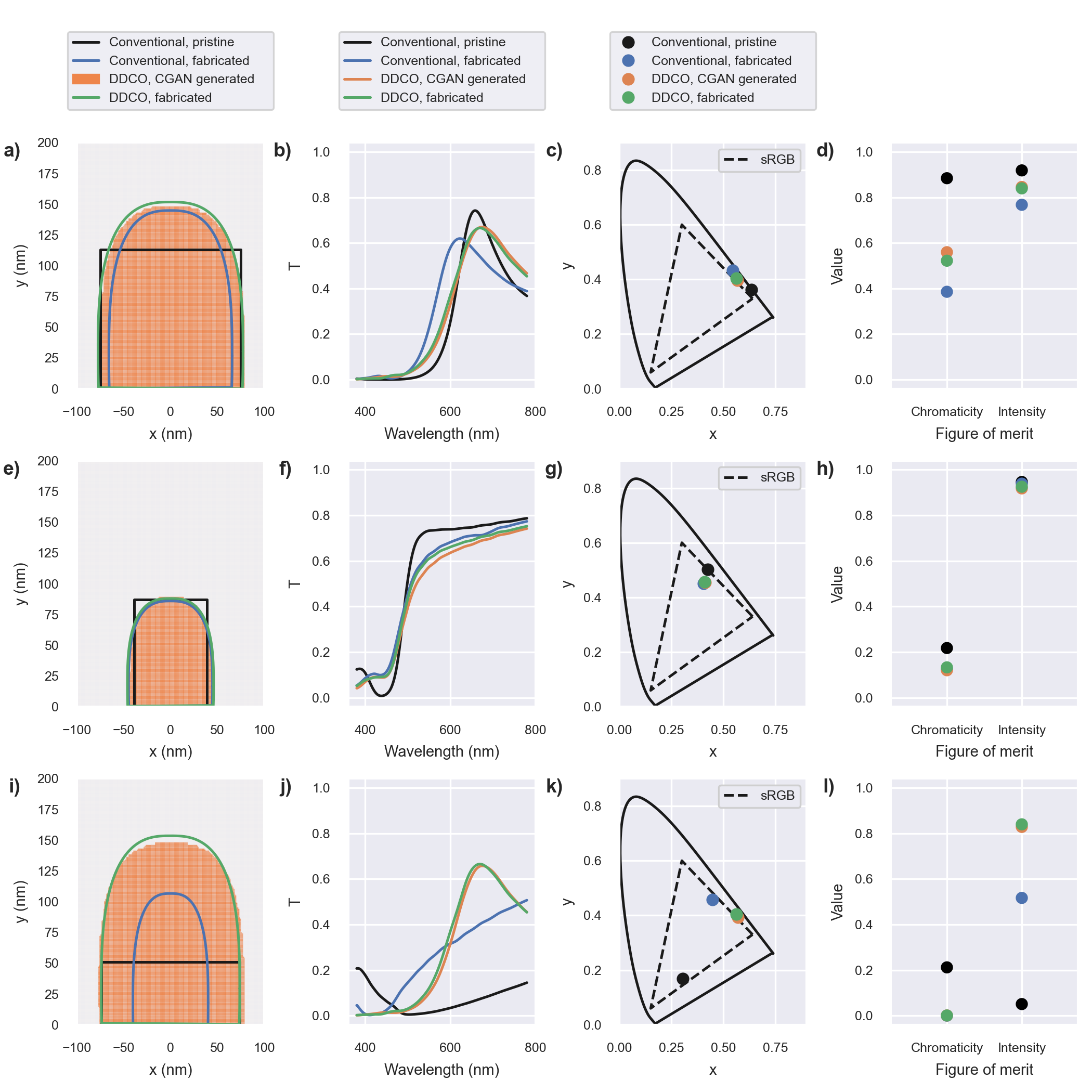


**Figure S6:** Grating optimization extended results. (a, e, i) Cross section of the different structures used in the optimization loops (pristine for conventional optimization, and CGAN generated for DCCO) and the corresponding profiles fabricated by virtual experiments for red and green optimizations. (b, f, j) Transmission spectra. (c, g, k) Transmitted colors assuming a D65 illuminant source compared to the standard RGB color space (dashed black line). (d, h, k) Figure of Merit (FOM) of the different structures depending on the optimization.

It is important to note that a simple metallic grating structure tend to behave like a long-pass filter, making it difficult to optimize green and blue structures. This is why in Figure S6, green optimization results appear like a long-pass filter and why the blue optimization is not returning any satisfying color filter design, with the conventional optimization returning a design with very poor chromaticity and poor transmission, while the DDCO design returned a design with high transmission but unusable chromaticity. To achieve green and blue color filters, dual resonances systems are recommended. Hence why we also provide examples of optimizations using grating on top of a waveguide in Figure S7.

However, all six examples illustrate that conducting optimization with DDCO and using generative network to simulate realistic structures reduce the discrepancies between the simulations profiles used in the optimization process (here in orange) and the potentially fabricated samples (here the virtually fabricated samples being drawn in green).

**Table S4:** Dimensions of optimized grating designs.

| Target color | Optimization | Period (nm) | Width (nm) | Height (nm) |
| --- | --- | --- | --- | --- |
| Red | Conventional | 200 | 150 | 113 |
| Red | DDCO | 200 | 150 | 120 |
| Green | Conventional | 200 | 78 | 87 |
| Green | DDCO | 200 | 50 | 136 |
| Blue | Conventional | 200 | 148 | 51 |
| Blue | DDCO | 200 | 150 | 135 |

The width and height are pre-annealing values, hence they the real profile may differ from those values, following the process presented in Data Generation section to adjust the structural parameters according to a non-linear model.


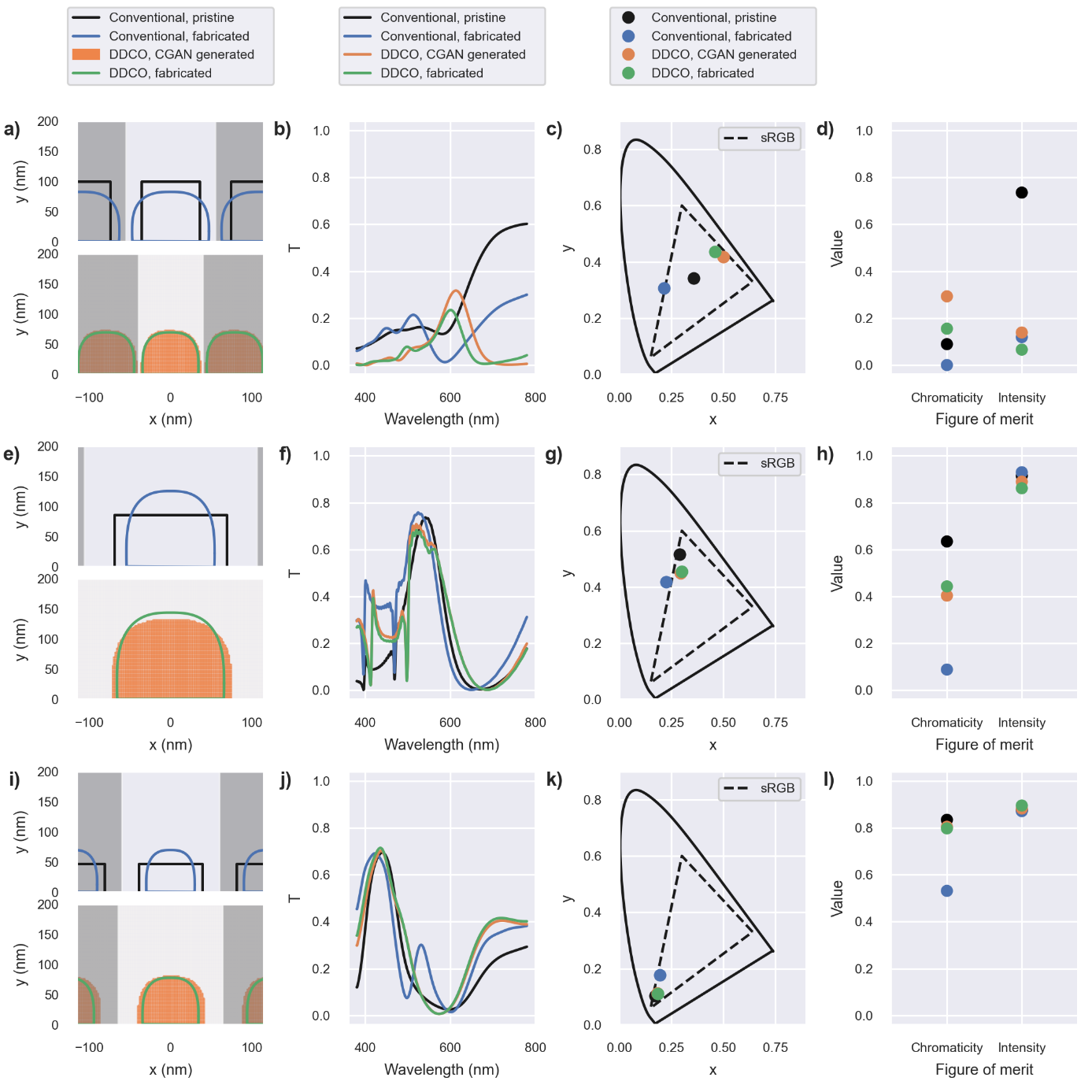


**Figure S7:** Grating optimization extended results. (a, e, i) Cross section of the different structures used in the optimization loops (pristine for conventional optimization, and CGAN generated for DCCO) and the corresponding profiles fabricated by virtual experiments for red, green, and blue optimizations. (b, f, j) Transmission spectra. (c, g, k) Transmitted colors assuming a D65 illuminant source compared to the standard RGB color space (dashed black line). (d, h, l) Figure of Merit (FOM) of the different structures depending on the optimization.

**Table S5:** Dimensions of optimized waveguide-grating designs. The grating’s width are fixed to have a fill factor of 0.65.

| Target color | Optimization | Period (nm) | Waveguide height (nm) | Grating height (nm) |
| --- | --- | --- | --- | --- |
| Red | Conventional | 91 | 58 | 116 |
| Red | DDCO | 79 | 450 | 50 |
| Green | Conventional | 213 | 255 | 86 |
| Green | DDCO | 230 | 252 | 115 |
| Blue | Conventional | 120 | 201 | 47 |
| Blue | DDCO | 129 | 203 | 58 |

The grating heights are the pre-annealing values, hence they the real profile may differ from those values, following the process presented in Data Generation section to adjust the structural parameters according to a non-linear model.

1. Radford, A., Metz, L., & Chintala, S. (2015). Unsupervised representation learning with deep convolutional generative adversarial networks. arXiv preprint arXiv:1511.06434. [↑](#footnote-ref-1)
